# Effects of chlorogenic acid on capacity of free radicals scavenging and proteomic changes in postharvest fruit of nectarine

**DOI:** 10.1371/journal.pone.0182494

**Published:** 2017-08-03

**Authors:** Yu Xi, Wenxiao Jiao, Jiankang Cao, Weibo Jiang

**Affiliations:** College of Food Science and Nutritional Engineering, China Agricultural University, No. 17 Qinghua Donglu, Beijing, PR China; University of PECS Medical School, HUNGARY

## Abstract

To study how chlorogenic acid affects changes of reactive oxygen species (ROS) and the proteins involved in ROS scavenging of nectarine during storage time, the fruits were treated with chlorogenic acid (CHA) then stored at 25°C for further studies. The CHA-treatment significantly reduced O_2_^-^· production rate, H_2_O_2_ content, and membrane permeability of nectarine fruit during storage. The key proteins related the nectarine fruit senescence during storage were identified by two-dimensional electrophoresis and MALDI-TOF/TOF. Level and enzymatic activity of peroxidase were reduced, while both the protein levels and the enzymatic activities of superoxide dismutase, glutathione reductase, glutathione-s-transferase and monodehydroascorbate reductase were enhanced in nectarine fruit treated with CHA. In addition, levels of several pathogen-related proteins were also enhanced by CHA-treatment. Taking together, the present study showed that CHA could influence changes in defense related proteins and reduced oxidative damage in nectarine fruit during postharvest ripening.

## Introduction

Nectarine, belonging to the *Rosaceae* family, is a typical climactic fruit. It originated from China and represents one of the most important species of the stone fruits [1]. Nectarine deteriorates rapidly after harvest and usually results in a short limited postharvest life [2]. During ripening, numerous biochemical, physiological and structural changes occur in the fruit.

Reactive oxygen species (ROS), including superoxide radicals (O_2_^-^·), hydrogen peroxide (H_2_O_2_) and hydroxyl radicals (HO·), are known to be products of stress response and inevitably generated via a number of normal metabolic pathways [3]. ROS can be deleterious to cellular functions. This continual cellular damage may cause lipid oxidation, protein oxidation, DNA strand breaking and base modification, and modulation of gene expression [4]. Therefore, ROS can play an important role in the progress of senescence and various senescence-associated disorders [5]. Peach fruit have been reported to accumulate O_2_^-^· production rate, H_2_O_2_ content, malonaldehyde (MDA) content and membrane permeability during postharvest ripening [6].

Chlorogenic acid (CHA) is a principle phenolic compound in nectarine fruit pulp [7] and has strong antioxidant activity [8], which is positively correlated with ROS scavenging ability in peach and nectarine fruit [9,10]. However, little is known about effects of polyphenols on fruit proteins [11]. In previous studies, we have demonstrated that exogenous CHA can significantly delay senescence of apple fruit [12]. In addition, studies show that CHA may affect activities of antioxidant enzymes, such as superoxide dismutase (SOD), catalase (CAT), ascorbate peroxidase (APX) and glutathione reductase (GR), which can play important roles in counteracting the toxicity of ROS [13].

Our previous studies have demonstrated that CHA could improve postharvest quality, influence antioxidant properties and reduce MDA content of nectarine fruit during postharvest ripening [14], however, it is still not fully explored the mechanisms underlying those effects of CHA, such as lacking the relevant information of proteomics.

To investigate the effects of CHA on proteins related to ROS scavenging in nectarine fruit, two-dimensional electrophoresis combing with analysis of MALDI-TOF/TOF were used in the present study. We demonstrated that CHA could influence changes in defense related proteins and reduced oxidative damage in nectarine fruit during postharvest ripening.

## Material and methods

### Plant material

Nectarine [*P*. *persica* (L.) Batsch, var. nectarine, c.v. Ruiguang] fruit at green mature stage were obtained from a commercial orchard in Beijing, China, and was selected for uniformity in shape, color, and size, and then were used for the experiments.

### Treatment

Nectarine fruit were randomly divided into two groups. One group was provided as the untreated control and the other group was infiltrated with 50 mg L^-1^ chlorogenic acid (3-O-caffeoylquinic acid, CHA) solutions under vacuum (-0.02 M Pa) for 2 min and kept in the solutions for additional 3 min at 25°C without vacuum. The control nectarines also were vacuum infiltrated with distilled water. Fruits were air dried, and then were stored at 25°C with 80–90% relative humidity. Samples were conducted at 0, 2, 4, 6, 8 days of storage, respectively and the sampled tissues were immediately powdered in liquid nitrogen and stored at -80°C until further analysis. Three replications were conducted in this experiment, and there were 60 fruits in each replicate.

### Assays of reactive oxygen species (ROS), MDA and membrane permeability

Superoxide radical (O_2_^-^·) production rate was determined according to [15] with some modifications. 1.0 g of pulp powders were homogenized with 1.0 mL of extraction solution [50 mM sodium phosphate buffer (pH 7.8), 1 mM EDTA, 2% polyvinylpyrrolidone (PVP, w/v) and 0.3% Triton X-100]. The homogenate was centrifuged at 10,000×g for 30 min at 4°C. 1.0 ml of the supernatant was mixed with 1.0 ml of 50 mM sodium phosphate buffer (pH 7.8) and 1.0 ml of 10 mM hydroxylamine hydrochloride. After incubation at 25°Cfor 1 h, 1 ml of 34 mM sulfanilic acid and 1 ml of 7 mM α-naphthylamine was added to the mixture for another 20 min at 25°C. The absorbance was measured at 530 nm for the measurement of O_2_^-^·. The O_2_^-^· production rate is expressed as mmol min^-1^ kg^-1^ fresh weight.

The hydrogen peroxide (H_2_O_2_) content was determined according to the method previously described [16]. 2.0 g of pulp powders were homogenized with 2.0 mL 0.1% (w/v) trichloroacetic acid. The homogenate was centrifuged at 10,000×g for 30 min at 4°C. Thereafter, 1.0 mL of the solution was mixed with 1.0 mL of 0.1% trichloroacetic acid, 1.0 mL 0.1 M sodium phosphate buffer (pH 7.0), and 2.0 mL 1 M potassium iodide. After incubation in dark for 1 h at 25°C, the absorbance of the supernatant was measured at 390 nm. The H_2_O_2_ content is expressed as mmol kg^-1^ fresh weight.

Membrane permeability was expressed as relative electrolyte leakage rate according to previous study [6].

### Enzyme assays

Peroxidase (POD, EC 1.11.1.7): 2.0 g of pulp powders were homogenized with 2.0 ml of 0.1 M sodium acetate buffer (pH 5.5) containing 0.34% PEG 6000 (w/v), 4% (w/v) polyvinypyrrolidone (PVP) and 1% (v/v) Triton X-100.

Superoxide dismutase (SOD, EC 1.15.1.1) and catalase (CAT, EC 1.11.1.6): 2.0 g of pulp powders were homogenized with 2.0 ml of 0.1 M sodium phosphate buffer (pH 7.5) containing 5% (w/v) PVP and 5 mM dithiothreitol.

Ascorbate peroxidase (APX, EC 1.11.1.11): 2.0 g of pulp powders were homogenized with 2.0 mL of 0.1 M potassium phosphate buffer (pH 7.5) containing 2% polyvinlpyrrolidone cross linked (PVPP), 1 mM ascorbic acid and 1 mM ethylene diamine tetraacetic acid (EDTA).

Glutathione reductase (GR, EC 1.6.4.2): 2.0 g of pulp powders were homogenized with 2.0 mL of 0.1 M sodium phosphate buffer (pH 7.5) containing 1 mM EDTA, 2 mM dithiothreitol (DTT).

Glutathione-s-transferase (GST, EC 2.5.1.18): 2.0 g of pulp powders were homogenized with 2.0 mL 0.2 M sodium phosphate buffer (pH 8.0), containing 1 mM EDTA, 4% (w/v) PVPP, 1mM DTT.

Monodehydroascorbate reductase (MDHAR, EC 1.6.5.4): 2.0 g of pulp powders were homogenized with 50 mM Tris-HCl (pH 7.8).

All the homogenates were then centrifuged at 10,000×g for 30 min at 4°C. The supernatants were used for the enzyme assays.

POD activity was determined by the increase in absorbance at 470 nm according to former research [17]. One unit (U) of POD activity is defined as the amount of enzyme that causes an increase in absorbance of 1 at 470 nm per minute.

SOD activity was determined by measuring its ability to inhibit the photoreduction of nitro-blue-tetrazolium (NBT) as described by previous researchers [18]. U of SOD activity is defined as the amount of enzyme that causes a 50% inhibition of NBT reduction at 560 nm.

CAT activity was measured by monitoring the decomposition of H_2_O_2_ at 240 nm following the method of previous study [19]. U of CAT activity is defined as the amount of enzyme that causes a decrease in absorbance of 1 per minute.

APX activity was determined as described in previous research [20]. The activity was calculated from change in absorbance at 290 nm. U of APX activity is defined as the amount of enzyme that causes a decrease in absorbance of 1 per minute.

GR activity was determined by the increase in absorbance at 334 nm due to former scientists [21]. U of GR activity is defined as the amount of enzyme that causes a decrease in absorbance of 1 per minute.

GST activity was determined by the increase in absorbance at 334 nm due to formation of s-(2, 4-dinitrophenyl) glutathione (DNP-GS) from 1-chloro-2,4-dinitrobenzene (CDNB) and GSH, according to former researchers [21]. U of GST activity is defined as the amount of enzyme that causes an increase in absorbance of 1 at 334 nm per minute.

MDHAR activity was determined by following the decrease in absorbance at 340 nm due to NADH oxidation, according to previous study [22]. U of MDHAR activity is defined as the amount of enzyme that causes a decrease in absorbance of 1 at 340 nm per minute.

The activity of each enzyme is expressed on a protein basis (U mg ^-1^ protein). Protein content in the enzyme extracts was determined according to the Bradford method [23], using bovine serum albumin (BSA, Sigma USA) as standard.

### Protein sample preparation

Briefly, 1.0 g of frozen sample was finely powdered in a mortar with liquid nitrogen and then homogenized with 150 μL ice-cold 1 M Tris (pH 11.2) and 30 mg PVPP. The homogenate was centrifuged at 10,000×*g* for 30 minutes, at 4°C.The supernatant was collected and dialyzed in 10 mM Tris (pH 7.5) overnight at 4°C, then was concentrated by lyophilization. Protein content was determined by Bradford method [23], using BSA (Sigma, USA) as standard.

### Two-directional electrophoresis and staining

After extraction, the proteins were solubilized in lysis buffer (7 M urea, 2 M thiourea, 4% (w/v) CHAPS, 1% (w/v) DTT and 0.5% (v/v) pH 3–10 IPG buffer). 1 mg proteins were applied to 17 cm pH 3–10 IPG strips, and isoelectric focusing was performed on a PROTEAN IEF system (Bio-Rad, USA) for a total of 116.4 kVh at 20°C. Then, the strips were equilibrated for two periods of 15 minutes with 1% (w/v) 1,4-dithiothreitol and 2.5% (w/v) iodoacetamide in equilibration buffer. Following equilibration, the strips were run on 12% home-made gels with a vertical set (Bio-Rad, USA). Then the gels were stained with 0.1% Coomassie Brilliant Blue R250. Three biological replicates were performed for each treatment and each biological replicate with three technical replicates.

### Image acquisition and data analysis

The stained gels were imaged by a Versdoc 3000 scanner (Bio-Rad, USA), and analyzed by PDQuest Version 8.0 (Bio-Rad, USA). Images were properly cropped and optimized, and then subjected to gel-to-gel matching with standard protein maps. The abundance of each protein spot was estimated by the percentage volume (% volume), i.e. the spot volume was normalized as a percentage of the total volume of all spots in the gel. Finally, the spots that changed more than 2-fold and passed the Student's t-test (*p* < 0.05) were considered as differentially abundant proteins.

### Protein in-gel digestion and identification by MALDI-TOF-TOF/MS

Method for protein in-gel digestion and identification by MALDI-TOF-TOF/MS was accorded to former researchers [24]. Tryptic peptide masses were analyzed by a 4700 MALDI-TOF/TOF Proteomics Analyzer (Applied Biosystems, USA). Proteins were identified by searching against the NCBInr database “Rosaceace” using an in-house MASCOT server v2.1 (Matrix Science, London). The subcellular localization prediction of 18 differentially abundant proteins was based on PSORT (http://wolfpsort.org).

### Statistical analysis

Data were evaluated by the analysis of variance (ANOVA) with Statistical Analysis System of SPSS Statistics 17.0 (SPSS Inc., Chicago, Illinois, USA). Significant differences were performed by Duncan’s new multiple range tests, where differences at *p* < 0.05 were considered as significant.

## Results

### Changes of ROS generation in nectarine fruit during storage and response to CHA

The O_2_^-^· production rate in nectarine fruit increased from 0.80±0.03 μmol g^-1^min^-1^ to 0.96±0.06 μmol g^-1^min^-1^ and the H_2_O_2_ content increased dramatically from 0.28±0.05 μmol g^-1^ to 2.17±0.33 μmol g^-1^ during storage at 25°C for 8 days. Treatment with CHA significantly reduced the ROS generation. The O_2_^-^· production rate or H_2_O_2_ content in the CHA-treated nectarine was only 0.77 μmol g^-1^min^-1^ or 1.36 μmol g^-1^ at end of the storage (Table 1). The membrane permeability in CHA treated fruit was about 91% of that in control at end of the storage.

**Table 1:** O_2_-· production rate (O_2_-·), H_2_O_2_ content, and membrane permeability of nectarine fruit during storage at 25°C and response to CHA.

Parameters	Treatments	Storage time (days)				
		0	2	4	6	8
O_2_-· (mmol kg^-1^min^-1^)	Control	0.80±0.03a	0.82±0.03ac	0.92±0.02b	0.92±0.02b	0.96±0.06bc
	CHA		0.79±0.03a	0.79±0.05a	0.86±0.01c	0.77±0.03a
H_2_O_2_ (mmol kg^-1^)	Control	0.28±0.05a	0.64±0.05b	0.94±0.05c	1.61±0.05d	2.17±0.33e
	CHA		0.28±0.13a	0.53±0.09b	0.58±0.17b	1.36±0.17f
Electrolyte	Control	45.20±0.03a	52.00±0.02b	55.72±0.03c	56.10±0.04d	58.60±0.02e
leakage (%total)	CHA		48.10±0.01f	50.20±0.01g	51.00±0.04b	53.30±0.03h

Data were expressed as mean±standard deviation (n = 3). Values of the same parameter with different letters are significantly different at *p* < 0.05

### The CHA caused enzymatic changes in nectarine fruit during storage

**Fig 1:**
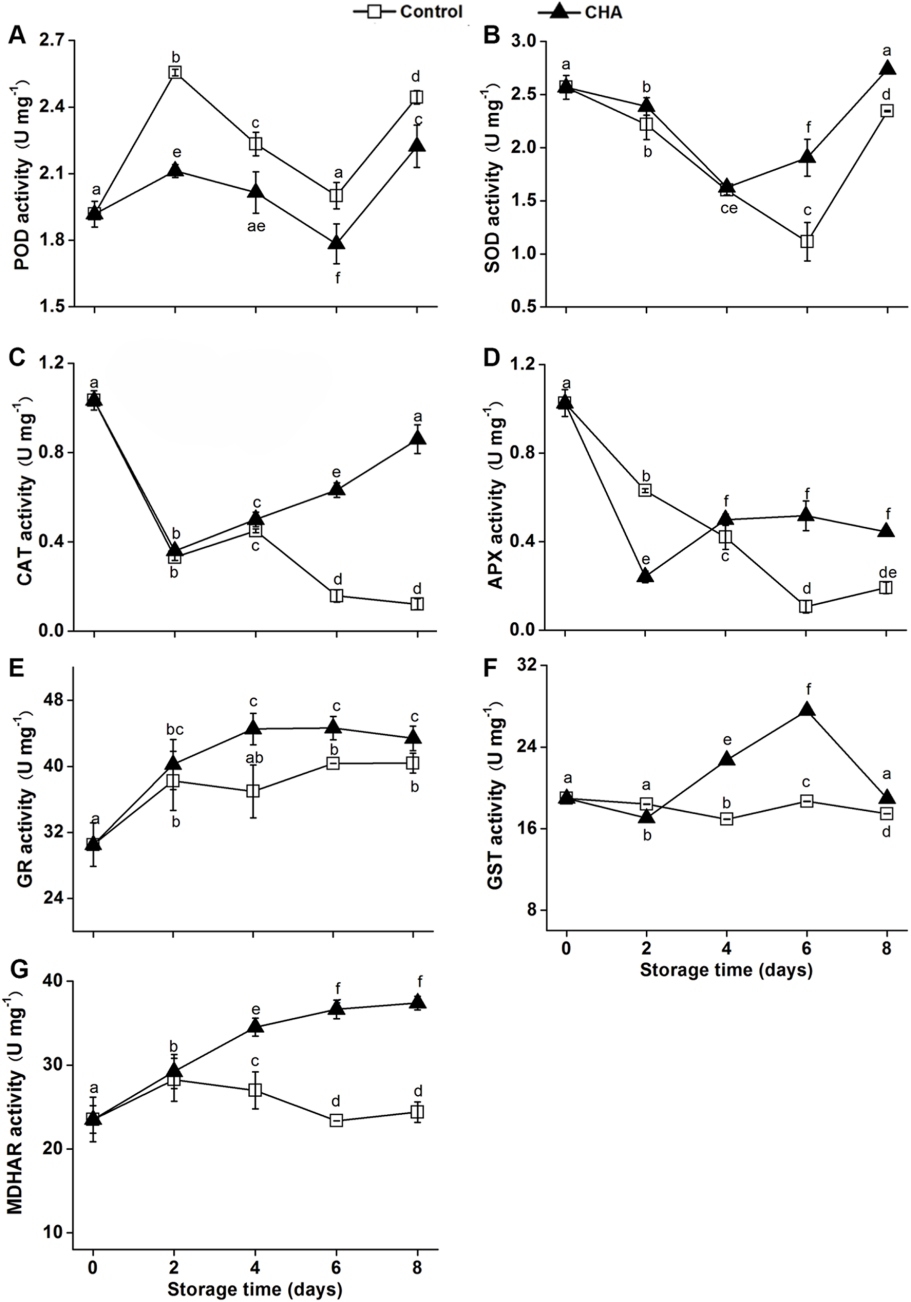
**POD (A), PPO (B), CAT (C), APX (D), GR (E), GST (F) and MDHAR (G) activities in nectarine fruit during storage at 25**°C **after CHA or water treatment (control).** Each value is the mean of three replicates. Vertical bars represent standard deviation of the mean. Values with different letters are significantly different at *p* < 0.05.

The postharvest treatment with CHA significantly reduced the total activities in nectarine fruit (Fig 1A) as well as the relevant protein level, such as phospholipid hydroperoxidase glutathione peroxidase (Table 2), as compared to the control fruit during the storage. Meanwhile the CHA treatment significantly enhanced enzymatic activates of superoxide dismutase (SOD), ascorbate peroxidase (APX), catalase (CAT), glutathione reductase (GR), glutathione-s-transferase (GST) and monodehydroascorbate reductase (MDHAR) in the fruit during the storage (Fig 1). Similar to the enzymatic variations, the CHA treatment enhanced the protein levels of Cu/Zn SOD, GR and S-transferase DHAR2-like (Table 2).

**Table 2:** Identification of different expressed 18 proteins in nectarine fruit pulp response to CHA treatment by MALDI-TOF-TOF/MS.

Spot no.^a^	Protein name and source	Accession no.^b^	Sequence coverage ^c^		Theo./Exp.^d^	Score	
			(%)	Mr(kDa) / pI			
1	small heat shock protein	AAR99375.1	43	17.38/17.51			268
	[Prunus persica]			5.98/8.39			
2	18.5 kDa class I heat shock protein-like	XP_008219998.1	32	18.139/26.75			356
	[Prunus mume]			5.84/8.35			
3	phospholipid hydroperoxide glutathione	XP_008238852.1	53	19.519/30.59			148
	peroxidase [Prunus mume]			4.79/4.98			
4	phospholipid hydroperoxide	XP_008238854.1	50	19.455/28.32			468
	glutathione peroxidase [Prunus mume]			5.11/4.99			
5	2-Cys peroxiredoxin BAS1,	XP_009349837.1	28	29.598/30.35			260
	chloroplastic-like [Pyrus x bretschneideri]			7.75/4.74			
6	Calmodulin	AF292108_1	46	16.894/20.75			128
	[Prunus avium]			4.11/4.19			
7	calmodulin	AF292108_1	46	16.894/17.52			360
	[Prunus avium]			4.11/4.36			
8	hypothetical protein	XP_007223083.1	34	41.974/47.09			250
	PRUPE_ppa006990mg [Prunus persica]			5.36/5.83			
9	glutathione reductase, cytosolic	XP_008224600.1	52	53.893/69.7			679
	[Prunus mume]			5.78/6.71			
10	CuZnSOD	AFP87312.1	57	15.485/18.84			482
	[Prunus persica]			5.6/6.26			
11	PREDICTED: glutathione S-transferase	XP_008233550.1	59	23.804/35.11			154
	DHAR2-like [Prunus mume]			6.1/4.29			
12	monodehydroascorbate reductase	XP_008241272.1	47	47.07/61.03			415
	[Prunus mume]			6.31/7.68			
13	hypothetical protein	EMJ03421.1	71	36.218/50.6			364
	PRUPE_ppa008531mg [Prunus persica]			4.78/5.02			
14	putative allergen Pru du 1.06B	ACE80949.1	63	17.413/20.76			114
	[Prunus dulcis x Prunus persica]			5.1/5.36			
15	major allergen Pru p 1	ABB78006.1	68	17.637/20.69			449
	[Prunus persica]			5.79/6.2			
16	pathogenesis related protein PR10	ABW99628.1	58	17.637/20.44			181
	[Prunus persica]			5.78/5.89			
17	putative allergen Pru p 1.06A	ACE80952.1	63	17.372/20.57			434
	[Prunus dulcis x Prunus persica]			5.1/5.04			
18	profilin	CAD37201.1	97	14.109/14.71			531
	[Prunus persica]			4.67/4.77			

a. Numbering corresponds to the 2-DE gel in Fig 1.

b. gi number from the NCBInr/EST database.

c. Percentage of predicated protein sequence with matched sequence.

d. Theoretical and experimental mass (kDa) and pI of identified proteins.

**Fig 2:**
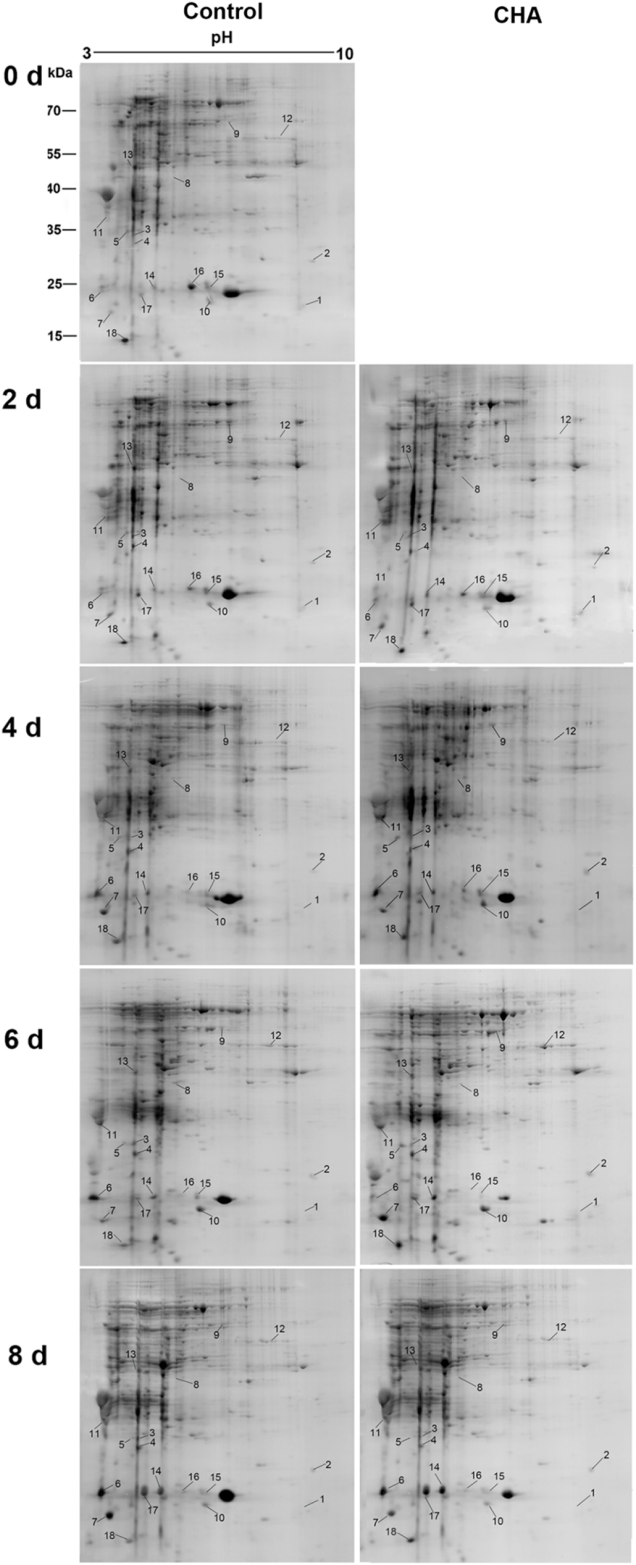
Identification of 18 proteins influenced by CHA in nectarine fruit by 2-DE and MALDI-TOF/TOF analysis.

**Fig 3:**
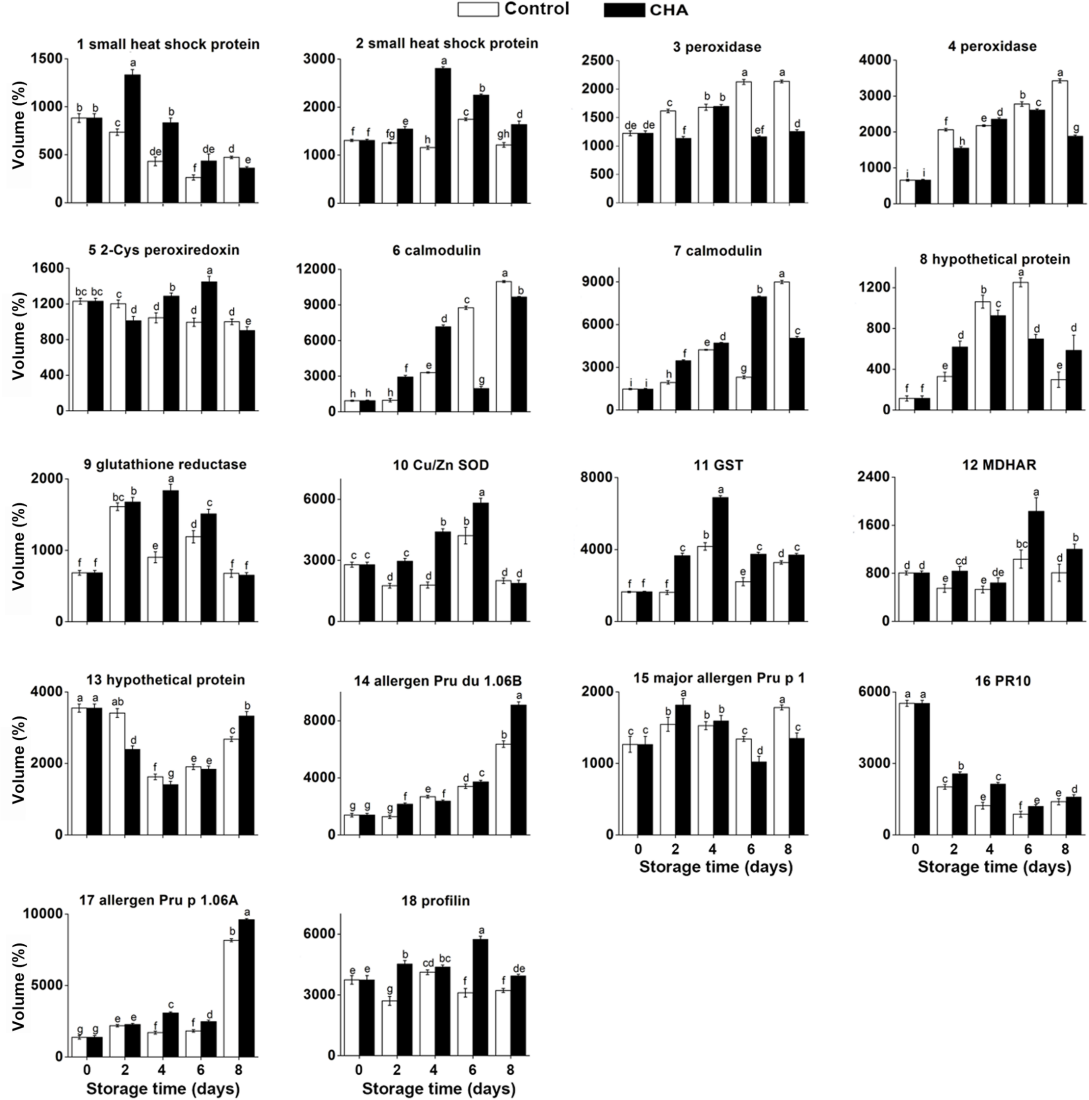
Accumulation of 18 proteins influenced by CHA in nectarine fruit during storage. Each value is the mean of three replicates. Vertical bars represent standard deviation of the mean. Values with different letters are significantly different at *p* < 0.05.

Our results showed that the abundance of 2-Cys peroxiredoxin BAS1, chloroplastic-like (2-Cys Prx, spot 5) (Table 2) decreased throughout the whole storage time and was also enhanced by CHA treatment (Figs 1–3).

**Fig 4:**
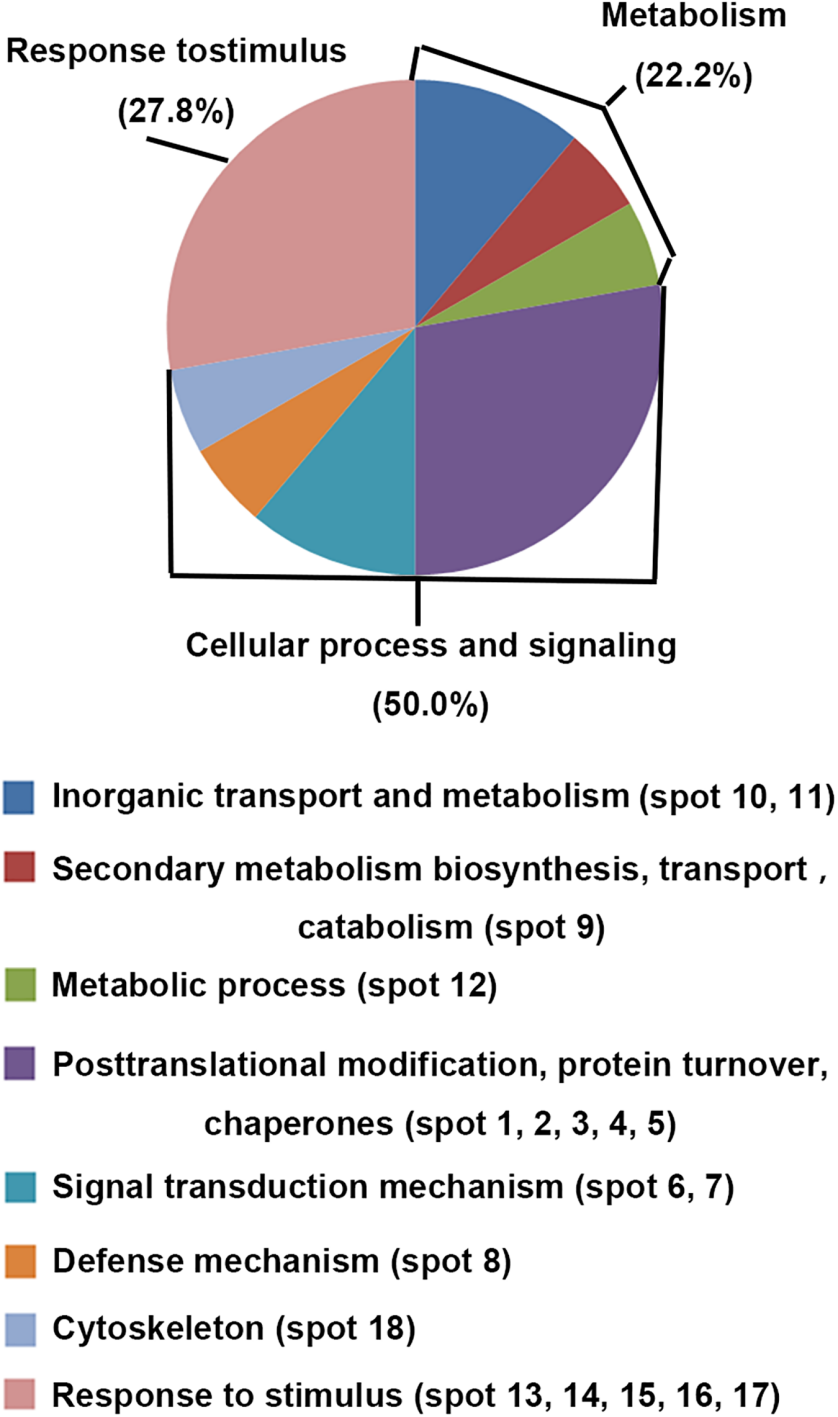
Classification and functional distribution of ripening related proteins in nectarine fruit identified by 2-dimentional electrophoresis and MALDI-TOF-TOF/MS. Protein species were categorized according to Gene Ontology annotation (http://www.ncbi.nlm.nih.gov/GO/) and eukaryotic orthologous groups (KOG, http://www.ncbi.nlm.nih.gov/COG/).

As shown in Fig 4, the 18 CHA-related proteins identified from nectarine fruit were classified according to Gene Ontology annotation (http://www.ncbi.nlm.nih.gov/GO/) and eukaryotic orthologous groups (KOG, http://www.ncbi.nlm.nih.gov/COG/). 50% of these proteins is involved in cellular process and signaling, including posttranslational modification, signal transduction mechanism, defense mechanism and cytoskeleton. Among these 18 proteins, 27.8% of them is involved in response to stimulus and 22.2% is involved in metabolism.

### Other proteins involving ROS scavenging

Two lowly expressed small heat shock proteins (sHSPs, spot 1 and 2) (Table 2) were identified in this study, which was both largely induced by CHA and kept stable during whole storage period (Figs 2–4).

Three structural proteins: calmodulin (spot 6, 7) and profilin (spot 18) (Table 2) were identified. At 4^th^ day and 6^th^ day of observation, calmodulin was remarkably induced by CHA treatment, and profilin of CHA group kept higher levels throughout the whole storage time than the control group (Figs 2 and 3).

Our results showed some allergen proteins and pathogen-related proteins were nearly all up-regulated by CHA during the observation. With CHA treatment, levels of putative allergen Pru p 1.06B (spot 14), PR 10 (spot 16), and putative allergen Pru p 1.06 A (spot 17) were all enhanced (Table 2, Figs 2 and 3). Likewise accumulation of Major allergen Pru p1 (spot 15) was significantly increased by CHA (Table 2, Figs 2 and 3).

Two hypothetical protein involved were successfully identified: hypothetical protein PRUPE_ppa006990mg (spot 8), hypothetical protein PRUPE_ppa008531mg (spot 13) (Table 2). It seemed that CHA treatment did not remarkably influence the accumulations of them (Figs 2 and 3).

### Correlation analysis

In this study, a significant positive correlation was found between protein expression accumulations of POD (spot 3), GR, MDHAR and their enzyme activities. As shown in Table 3, the correlation for POD (spot 3) *vs*. POD activity, GR *vs*. GR activity and MDHAR *vs*. MDHAR activity were 0.410 (*p*<0.05), 0.407 (*p*<0.05) and 0.526 (*p*<0.01), respectively. Although it was not significant, a positive correlation between protein expression accumulations of POD (spot 4) and GST and their enzyme activities was also existed.

**Table 3:** Correlation coefficients (r) among accumulation of POD, GR, MDHAR, GST and SOD expressions and their enzyme activities from nectarine pulp.

	Enzyme activities				
Accumulations of					
protein expression	POD	GR	SOD	GST	MDHAR
POD (spot 3)	0.410*				
POD (spot 4)	0.260				
GR		0.407*			
Cu/Zn SOD			-0.459*		
GST				0.343	
MDHAR					0.526**

* means correlation is significant at the *p*<0.05 level (2-tailed)

** means correlation is significant at the *p*<0.01 level (2-tailed).

### *In silico* subcellular locations

According to the WOLF PSORT database (http://wolfpsort.org/), the 18 identified proteins are assigned to five categories (Table 3), including cytoplasm (10, 55.6%), chloroplast (3, 16.7%), nuclear (3, 16.7%), endoplasmic reticulum (1, 5.6%), extracellular (1, 5.6%) (Table 4). Therefore, most of these proteins are predicted to locate in cytoplasm.

**Table 4:** Protein subcellular location prediction of the 18 proteins influenced by CHA in nectarine fruit according to PSORT (http://wolfpsort.org).

Spot no.	Protein name	Site	ID	Identity (%)
1	small heat shock protein	Cytoplasm	HS11_HELAN	70.70
2	18.5 kDa class I heat shock protein-like	Cytoplasm	HS11_SOYBN	74.53
3	probable phospholipid hydroperoxide glutathione peroxidase	Chloroplast	FER2_EQUTE	15.88
4	probable phospholipid hydroperoxide glutathione peroxidase	Chloroplast	IAA4_ARATH	13.37
5	2-Cys peroxiredoxin BAS1, chloroplastic-like	Chloroplast	ILV5_PEA	13.60
6	calmodulin	Nuclear	At3g54990.1	15.38
7	calmodulin	Nuclear	At3g54990.1	15.38
8	hypothetical protein PRUPE_ppa006990mg	Endoplasmic reticulum	At4g24520.1	13.87
9	glutathione reductase, cytosolic	Cytoplasm	GSHR_PEA	84.74
10	CuZnSOD	Cytoplasm	SODC_PEA	79.61
11	PREDICTED: glutathione S-transferase DHAR2-like	Cytoplasm	SODC_PANGI	14.62
12	monodehydroascorbate reductase	Extracellular	AMY1_HORVU	12.42
13	hypothetical protein PRUPE_ppa008531mg	Cytoplasm	At5g59290.1	13.41
14	putative allergen Pru du 1.06B	Cytoplasm	BV1A_BETVE	56.88
15	major allergen Pru p 1	Cytoplasm	BV1M_BETVE	62.50
16	pathogenesis related protein PR10	Cytoplasm	BV1M_BETVE	62.50
17	putative allergen Pru p 1.06A	Cytoplasm	BV1A_BETVE	55.63
18	profilin	Nuclear	DI13_CHLRE	14.50

## Discussion

Loss of membrane integrity and function under various stress conditions, including senescence during storage of nectarine fruit is generally associated with excessive accumulation of ROS including O_2_^-^·, H_2_O_2_ and hydroxyl radical. So quenching ROS should contribute to delay senescence of nectarine fruit after harvest. Our results showed that when nectarines were treated with CHA, the O_2_^-^· production rate and H_2_O_2_ content in the fruit were remarkably lower than the control group (Table 1). These could be due to increase in activities of the enzymes of eliminating ROS, such as CAT and SOD, as well as being partly caused by the increase of CHA content in the fruit after the treatment with CHA.

POD is well known as indicators of quality deterioration such as flavor loss and various biodegradation reactions [25]. Our result showed that treatment with CHA could reduce both enzymatic activity of POD and protein level of POD in nectarine. Similarly, other study has reported that apple polyphenols, which is mainly composed of CHA and several other polyphenols, can significantly inhibit POD activity [11]. These suggest that the effect of inbiting POD may partily account for CHA improving quality of nectarine fruit.

The enzymes, including SOD, CAT, APX, GR, GST and MDHAR, can participate in direct or indirect scavenging of ROS, and have been implicated in various senescence-related stress responses in plants [26]. 2-Cys peroxiredoxin (2-Cys Prx, spot 5) is highly conserved, abundant antioxidant enzyme that can catalyze the breakdown of peroxides to protect cells from oxidative stress [27] Our results showed that CHA treatment could enhance accumulation of 2-Cys Prx, and this may contribute to decrease ROS of fruit.

In the present study, higher activities of SOD, CAT, APX, GR GST and MDHAR in CHA-treated nectarine fruit could be beneficial in scavenging ROS and contributing to inhibition of senescence during storage. In agreement with previous study, our study also showed that antioxidant enzymes played the most important role in quenching ROS.

Previous studies have reported that sHSPs could be induced by various postharvest treatments during fruit ripening. For instance, treating tomato fruit with methyl jasmonate or methyl salicylate can induce gene expression of sHSPs and alleviate chilling injury of tomato [28]. In present study, we found that CHA could induce accumulation of sHSPs (Fig 3) and this might partly account for decrease of ROS in nectarine fruit with application of CHA.

The location analysis of differential proteins indicates that CHA treatment regulating functions differed on the subcellular level during fruit ripening. However, further studies are in need to interpret the protein abundance differences, to investigate the functional significance of differentially abundant proteins in whole cells and to figure out the relationship between differential proteins and organelles.

In conclusion, the O_2_^-^· production rate, H_2_O_2_ content, and membrane permeability in nectarine fruit increased during storage at 25°C and were significantly reduced by CHA treatment. Proteomics studied by 2-DE and MALDI-TOF-TOF/MS showed that levels of POD were reduced, while antioxidant enzymes including SOD, GR, GST, MDHAR, 2-Cys Prx were both enhanced. And correlation analysis showed that there was a positive correlation between levels of antioxidant proteins and their enzyme activities. Enzymatic activities of catalase and ascorbate peroxidase which are also important antioxidant enzymes were also enhanced by CHA-treatment. Taking together, the present study showed that CHA could influence changes in ROS of nectarine fruit during storage. Our findings should provide new insights into the mechanisms underlying endogenous polyphenols in regulation of postharvest ripening and senescence of climacteric fruits.

## Supporting information

S1 Fig Closer views of some proteins of nectarine fruit. (TIF)
