# Supplementary figures and images for: Effects of chlorogenic acid on capacity of free radicals scavenging and proteomic changes in postharvest fruit of nectarine
Source: PLoS One. 2017 Aug 3;12(8):e0182494. doi: 10.1371/journal.pone.0182494 (PMC5542658; doi:10.1371/journal.pone.0182494)

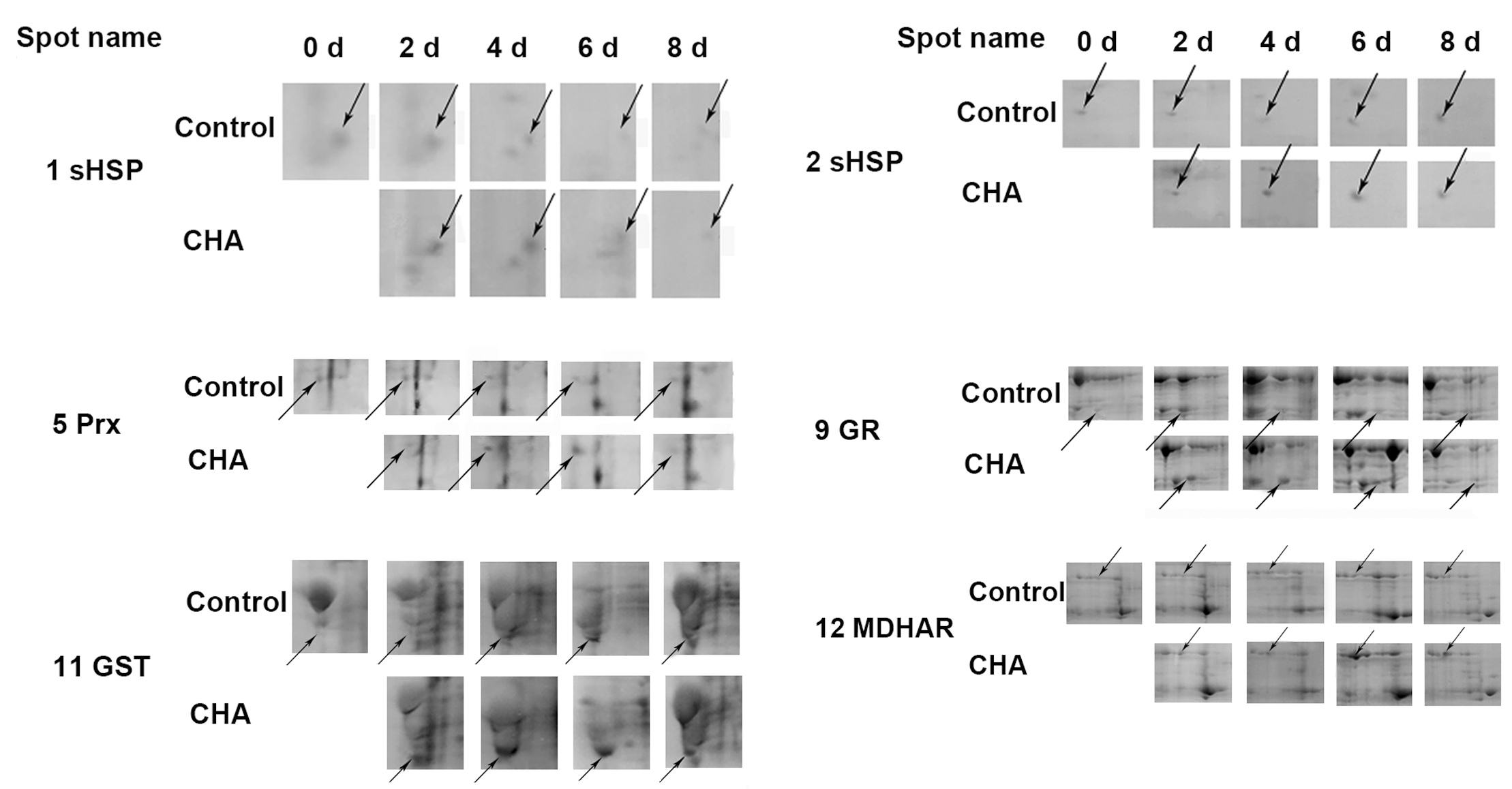

Supplement: S1 Fig — (TIF) [file pone.0182494.s001.tif]
